# The timeline of information exchange: a service evaluation of London Ambulance Service NHS Trust’s front line communication and emergency response to Exercise Unified Response

**DOI:** 10.29045/14784726.2020.12.4.4.40

**Published:** 2020-03-01

**Authors:** Jordan Nunan, Samantha Palfreyman-Jones, Rebecca Milne, Alison Wakefield

**Affiliations:** University of Portsmouth: ORCID iD: https://orcid.org/0000-0002-6187-003X; London Ambulance Service NHS Trust; University of Portsmouth: ORCID iD: https://orcid.org/0000-0002-4542-8495; University of Portsmouth: ORCID iD: https://orcid.org/0000-0002-1553-9178

**Keywords:** body-worn cameras, communication, multi-agency response

## Abstract

**Introduction:**

Exercise Unified Response, Europe’s largest major incident training exercise to date, provided a rich environment for the emergency services to test their multi-agency crisis response capabilities. Supported by the London Ambulance Service NHS Trust, this service evaluation examined London Ambulance Service NHS Trust front line communication and decision-making via body-worn camera footage.

**Methods:**

Twenty London Ambulance Service NHS Trust front line responders and evaluators were each equipped with a body-worn camera during Exercise Unified Response. The service evaluation aimed to: (a) produce timelines of the London Ambulance Service NHS Trust’s response in order to identify key events and actions during the ‘golden hour’ (the crucial first hour in the care of trauma patients), the proceedings of command meetings and the multi-agency response; and (b) develop recommendations for future training and evaluations.

**Results:**

The service evaluation identified that, within the golden hour, London Ambulance Service NHS Trust first responders rightly and rapidly declared the event a major incident, requested resources and assigned roles. Triage crews were tasked quickly, though it was identified that their efficiency may be further enhanced through more detailed triage briefings prior to entering the scene. The command meetings (led by the Metropolitan Police) lacked efficiency, and all agencies could make more effective use of the multi-agency shared radio network to address ongoing matters. Finally, London Fire Brigade and London Ambulance Service NHS Trust teams demonstrated clear communication and co-ordination towards casualty extraction.

**Conclusion:**

Successful multi-agency working requires clear communication, information sharing and timely command meetings. It is recommended that Joint Emergency Services Interoperability Principles multi-agency talk groups should be utilised more frequently and used to complete a joint METHANE report. In addition, training in areas such as communication skills and detailed briefings will enhance the front line response. Finally, body-worn cameras are shown to be an effective service evaluation tool, as a basis for promoting best practice as well as highlighting areas for future training and evaluations.

## Introduction

**Figure 1:**
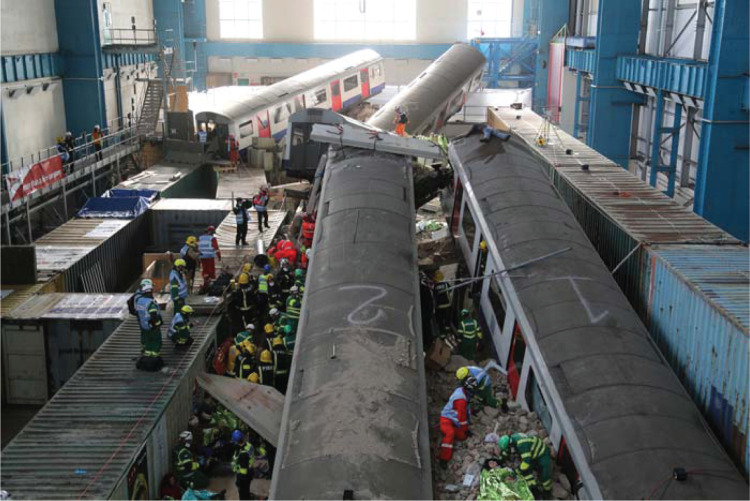
Figure 1. Inner cordon of Exercise Unified Response live simulation major incident site (London Fire Brigade, 2016).

In 2016, Exercise Unified Response (EUR), Europe’s largest major incident training exercise to date, provided a rich environment for the emergency services to test their multi-agency crisis response capabilities. The live simulation, which lasted four days, consisted of a building collapse replicating Waterloo train station, involving 4000 responders and 2500 casualties (see Figure 1) (London Fire Brigade, 2017), and was the first exercise to test the Hazardous Area Response Team (HART) national mutual aid response to this type of protracted major incident. The exercise involved all the emergency services as well as members of the public who acted as the casualties. The present service evaluation was independently conducted alongside EUR’s evaluation report (see London Fire Brigade, 2017) and focused on day one of EUR, which comprised 163 London Ambulance Service NHS Trust (LAS) staff and seven NHS Trust evaluators.

Training in a realistic simulated environment provided a safe environment for trainees to gain experience and develop a skillset ready for real incidents (Alison et al., 2013; Rouse, 1991). Controlled training environments therefore permit experimental control to evaluate individuals and inter-agency and multi-agency working (Alison et al., 2013). Such training is necessary to ensure resilience for when major incidents occur, especially as LAS has experienced 12 major incidents in the past five years. These major incidents included terrorist incidents such as the Parsons Green station bombing, Finsbury Park mosque attack, London Bridge attack and Westminster Bridge attack.

**Figure 2:**
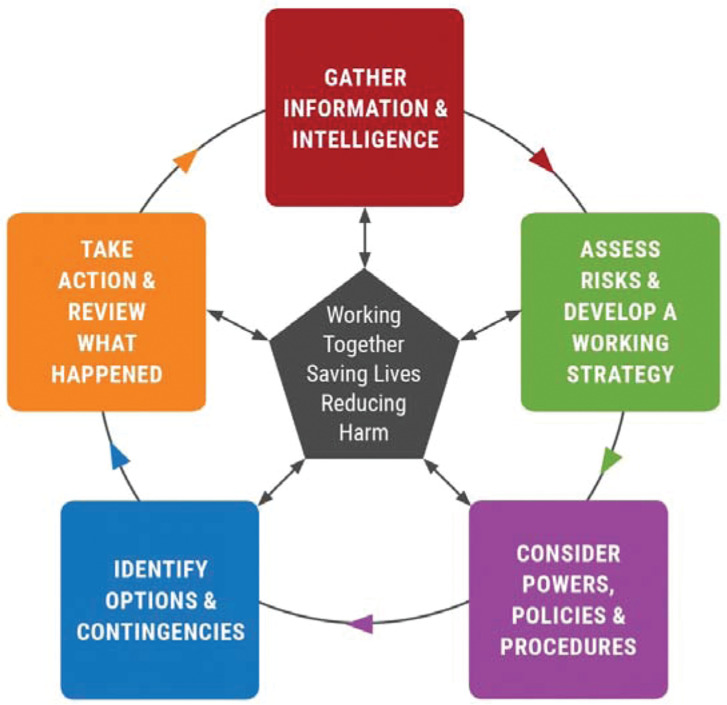
Figure 2. Joint Emergency Services Interoperability Principles (2015) multi-agency decision-making model.

In the course of a major incident, the collection and dissemination of reliable information is crucial to an effective and efficient multi-agency response (Allen, Karanasios, & Norman, 2014). Decisions based on incomplete or unreliable information are likely to result in poor quality decisions, leading to unacceptable outcomes in an environment where human life is at risk (Barrett & Smith, 2017; Power, 2017; Power, Boulton, & Brown, 2018). The techniques used to elicit and share information are, therefore, key to the success of the multi-agency response (Waring, Alison, & Humann, 2017). However, a number of factors may impact upon this process, such as (a) the sources of the information, and (b) the communication methods used to gather the information (e.g. open, closed or leading questions). The government’s Joint Emergency Services Interoperability Principles (JESIP) (Joint Emergency Services Interoperability Principles, 2015) programme, which was a two-year project seeking to improve the way in which the police, fire and rescue and ambulance services work together when responding to major multi-agency incidents, provided national guidance to help improve multi-agency co-ordination at major incidents. JESIP was established to address commonality across agencies for operational and command procedures, provide clarity regarding specialist resources and mitigate misunderstanding when sharing information and differing risk thresholds (see Figure 2; Joint Emergency Services Interoperability Principles, 2015).

However, limited guidance is provided to first responders on *how* to elicit information. In the emergency medical setting, the primary focus on the preservation of life may mean that reduced emphasis is placed on the collection of information that is not medically relevant. Furthermore, the psychological and physical well-being of the casualty may inhibit the collection of information, due to the possible onset of trauma and the unconscious state of some casualties. Since the reliability of the information collected will only be as reliable as the techniques used to elicit that information, it is important that communication strategies are set alongside witness care strategies and that the impact of trauma is managed within this triage process (see Risan, Binder, & Milne, 2016; Smith & Milne, 2018).

The aims of this service evaluation, undertaken with the support of LAS, were to examine front line communication and decision-making, both within and across LAS teams, and LAS’s emergency response. Specifically, it set out to: (a) produce timelines of key stages of the LAS response in order to identify key events and actions during the ‘golden hour’ (the first hour after injury, regarded as critical to the care of trauma patients), the proceedings of command meetings and the multi-agency response; and (b) develop recommendations for future training and evaluation opportunities.

## Methods

In order to conduct the service evaluation, a methodology of overt, structured observation by means of body-worn video footage was adopted from Nunan et al. (2016), which looked at front line communication, and the handover of information over time, captured by body-worn cameras (BWCs) attached to London Fire Brigade (LFB) front line responders during EUR. Specifically, 20 LAS emergency front line responders and evaluators, as listed in Table 1, were each equipped with a BWC, capturing a total of 233 clips of between 0.3 and 30.0 minutes long, with a mean length of 22.5 minutes, accumulating 92.5 hours from day one of EUR. Those equipped with BWCs were chosen due to their role and informed consent, in order to reflect a wide spread of roles, responsibilities and rank in the footage. This was the first time LAS front line responders had worn BWCs.

**Table 1.** Breakdown of London Ambulance Service NHS Trust staff members equipped with body-worn cameras.

LAS role	Definition of LAS role	BWC
NHS trust evaluator	The evaluators were subject matter experts in their chosen fields who conducted separate evaluations for LAS.	7
HART	HART comprises paramedics who are specially trained to work in hazardous areas and environments. These include: USAR; SWAH; CBRNe materials; inexpiable atmospheres requiring the wearing of breathing apparatus, gas tight suits and PRPS; and SRT.	3
MRU	Qualified paramedics who respond to incidents on a motorbike.	1
Paramedic	HCPC registered paramedic.	1
Command meeting	Command meetings are attended by incident commanders (operational, tactical or strategic). These could be at the incident site or remote in a designated place. They can be multi- or single-agency and at major incidents they will be a combination of the two.	1
MERIT	MERIT teams are made up of doctors and paramedics who have specialist training in trauma, usually through a rotation on London Air Ambulance. They are part of London’s pre-determined response to a major incident.	1
CCS medical lead	The CCS medical lead role is currently carried out by an APP or doctor. It involves overseeing the treatment of all patients in the CCS and includes deciding which patients leave the scene first.	1
ISO	The operational platform role came about during EUR. It is not a role included in London’s major incident plan. Due to the nature of the incident and the distance required for patients to exit the train station and receive more treatment, a member of HART was installed at the entrance to the platform to keep track of all responders, co-ordinate equipment, record casualty figures exiting the platform, ensure responders had the correct PPE to enter the area and keep a map of locations of the casualties.	1
Tactical advisor ISO	Tactical advisors are EPRR officers and a number of HART commanders who offer tactical and specialist advice either on the phone or on scene at any complicated calls, not just major incidents.	1
Operational medic	Operational medics manage the initial activity at the scene and are a representative of the tactical medic on scene.	1
Control room	The EOC is where all the 999 calls for the LAS come into. There are two control rooms in London, each with a special operations room attached to it. This is where a number of EOC staff and commanders locate to in a major incident.	1
Initial triage team	The initial triage team is the first set of responders to arrive on scene. The team enters the incident and carries out rapid triage, performing only basic airway manoeuvres and catastrophic haemorrhage control. There will always be a minimum of two per team and they are never out of each other’s line of sight.	1

Note: APP = Advanced Paramedic Practitioner; BWC = Body-Worn Camera; CBRNe = Chemical, Biological, Radiological, Nuclear and Explosive; CCS = Casualty Clearing Station; EOC = Emergency Operation Centre; EPRR = Emergency Preparedness Resilience and Response; EUR = Exercise Unified Response; HART = Hazardous Area Response Team; HCPC = Health and Care Professions Council; ISO = Incident Support Officer; LAS = London Ambulance Service NHS Trust; MERIT = Medical Emergency Response Incident Team; MRU = Motorcycle Response Unit; PPE = Personal Protective Equipment; PRPS = Powered Respiratory Protection Suit; SRT = Swiftwater and Flood Rescue Technician; SWAH = Safe Working at Height; USAR = Urban Search and Rescue.

The cameras were the sole source for data collection and were employed in order to explore the technology’s capabilities for LAS staff as well as for the purpose of capturing front line communication between and across agencies. From the body-worn footage, timelines were generated by recording all communicative and decision-making incidents into a spreadsheet. Each BWC was assigned the title of the LAS staff’s role (e.g. initial triage team, see Table 1) and all incidents were timestamped by the BWC’s display. Although the sample size was reasonable for the present service evaluation, in order to extrapolate the findings beyond those equipped with BWCs and those captured in the footage, more data may increase the confidence of the discussions. That said, 20 BWCs from one day of training captured 92.5 hours of footage.

## Results

The service evaluation focused on day one of EUR in order to capture the initial front line communication and decision-making in response to the major incident in three main contexts: during the ‘golden hour’, during command meetings and for multi-agency co-ordination. The findings in each of the three contexts are discussed in turn.

### Key events and actions during the ‘golden hour’

**Figure 3:**
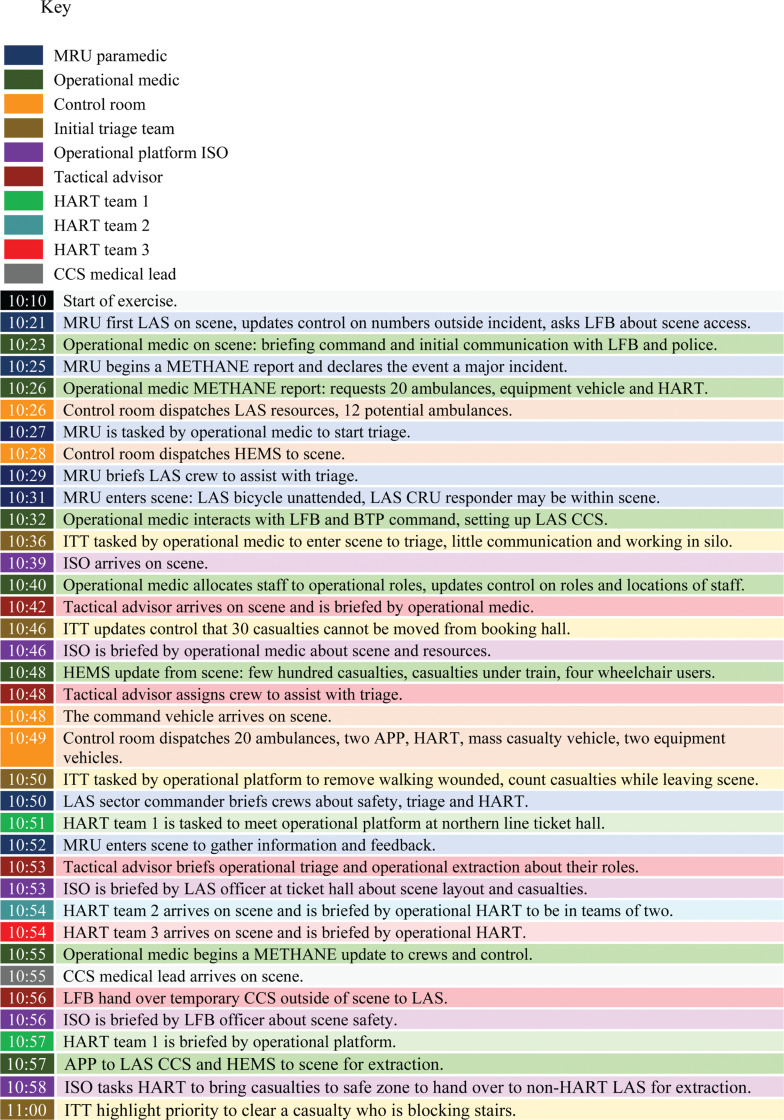

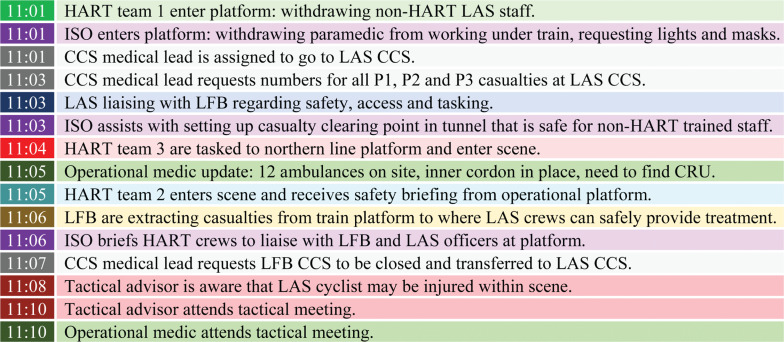
Figure 3. Timeline of ‘golden hour’.

Note: APP = Advanced Paramedic Practitioner; BTP = British Transport Police; CCS = Casualty Clearing Station; CRU = Cycle Response Unit; HART = Hazardous Area Response Team; HEMS = Helicopter Emergency Medical Service; ISO = Incident Support Officer; ITT = Initial Triage team; LAS = London Ambulance Service NHS Trust; LFB = London Fire Brigade; MRU = Motorcycle Response Unit.

Figure 3 displays a timeline of events throughout the ‘golden hour’. With the exercise having started at 10:10, the motorcycle response unit (MRU) paramedic was first on the scene within 11 minutes (10:21). A logistical delay notifying LAS that the exercise had started held up the response of the first ambulance. As first on scene, the MRU initiated a METHANE report and declared the event a major incident (10:25). They were shortly followed by the operational medic who, on arrival, requested equipment and specialist LAS teams to the scene (10:26). A first responder’s initial METHANE report is a vital opportunity to gather reliable information in a timely manner, covering: **m**ajor incident declared; **e**vent location; **t**ype of incident; **h**azards; **a**ccess; **n**umber, type and severity of casualties; and **e**mergency services present and required, which should subsequently inform the control room of appropriate resources to dispatch (10:26).

At the initial triage briefing before entering the scene (10:27), LAS crews were quickly tasked with triaging casualties (10:27–10:48), with an immediate focus on preserving life and assessing and reporting back the number of casualties at the scene. The initial triage briefing did not instruct them to count the number of casualties triaged by priority level (observed at 10:36), which was addressed in a second triage briefing at 10:50, meaning that one LAS officer was tasked with re-triaging casualties leaving the scene to provide an ongoing assessment of the casualties.

At 10:31 the MRU responder identified a cycle response unit (CRU) bicycle that had been left unattended outside the scene entrance, raising questions about the CRU responder’s welfare. This information was acted upon by the operational medic at 11:05. Although the CRU responder would have been an obvious casualty within the scene due to their uniform, the acknowledgement of the unattended bicycle at 10:31 did not appear to have reached the operational medic until 11:05.

The operational medic asked for the casualty clearing station (CCS) to be set up at 10:32, with a Helicopter Emergency Medical Service team reporting an approximation of a few hundred casualties, including a number of casualties under the train and four wheelchair users (10:48). This information was shared with the operational medic who updated their METHANE report accordingly (10:55), informing the response regarding equipment, staffing and planning for casualty extraction. While the CCS was in the process of being set up, LFB created a temporary casualty station outside the scene entrance to serve as a primary triage point before the CCS was operational and casualties could be transferred there. LFB handed over control of the temporary CCS to LAS at 10:56, with LAS requesting the closure of the LFB temporary CCS and transfer of casualties to the LAS CCS at 11:07.

At 11.01 the operational platform incident support officer (ISO) declared the scene to be a HART operation and, once the scene was declared a HART response, non-HART LAS responders were required to withdraw from the scene for safety reasons. For example, the operational ISO withdrew non-HART paramedics from working under the train (11:01).

In summary, within the golden hour, LAS first responders quickly and correctly declared the event a major incident, requested resources and assigned operational roles. Triage crews were swiftly tasked by the MRU paramedic, the CCS was established by the operational medic and the scene was confirmed as requiring a HART-led response. The vast array of actions undertaken within the first hour on scene, as laid out in Figure 3, exemplify the complexity and importance of the actions taken at this stage of a major incident.

### The proceedings of command meetings

Command meetings facilitate a multi-agency approach, allowing the emergency services and local responding agencies to collectively share information regarding aims, objectives, risks and resources. This is to enable agencies to work towards a shared superordinate goal, while also undertaking unique, agency-specific sub-goals (Waring et al., 2017). Inputs need to be thorough but also focused, equipping the on-scene commanders (operational and tactical officers) to return to the scene and feed-back the updates from the command meeting into their own organisations’ chain of command and front line responders. Hence, effective information sharing is fundamental to developing accurate situation awareness, informing planning (Klein, Calderwood, & Clinton-Cirocco, 1986) and ensuring a co-ordinated response (Davison, Hollenbeck, Barnes, Sleeman, & Illgen, 2012).

**Figure 4:**
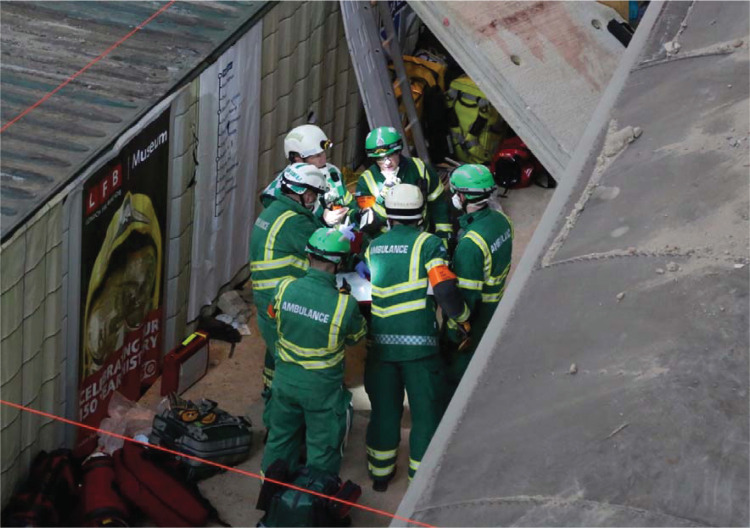
Figure 4. Hazardous Area Response Team (HART) briefing on platform (London Fire Brigade, 2016).

Throughout day one, the LAS operational medic provided regular METHANE and IRIMAC (**i**nformation, **r**isk, **i**ntention, **m**ethod, **a**dministration and **c**ommunication) reports to the tactical and strategic officers (see Figure 4). IRIMAC reports differ to METHANE reports as they refer to the operational planning and the assignment of responsibilities of staff rather than solely focusing on gathering information about the incident. Table 2 outlines the proceedings of the command meetings which took place throughout day one of EUR, highlighting some of the key activities and events.

**Table 2.** Command meetings.

**Tactical meeting (11:10)** The meeting was led by the police tactical commander and attended by representatives of all emergency services and local agencies at scene. The police, LFB and LAS representatives provided brief updates on their progress from the first hour. The LAS representatives reported resources at the scene (i.e. ambulances and specialist teams). The LFB represented provided a safety briefing regarding the stability of the scene. It was notable that multiple individuals from each agency attended the first tactical meeting (11:10).
**Tactical meeting (12:00)** LAS stated 12 ambulances on site and hospitals in use and announced that an LAS CRU responder was found deceased within the scene. Police stated that no emergency services personnel should interact with media unless briefed by a police media officer and permission had been granted by their own organisation.
**Tactical meeting (13:31)** Additional safety briefings were provided by LFB regarding asbestos and potential hospital contamination. Police state the importance of contacting DVI and the procedures when discovering a deceased casualty on scene. LAS request assistance from other services to extricate casualties to the CCS. Police and LFB assign further resources to be tasked by LAS regarding casualty extraction.
**Mass casualty meeting (16:37)** Storage space across London for deceased casualties was discussed. LAS briefed coroner with casualty numbers and that four deceased patients were alive when initially moved.
**Operational meeting (17:31)** All agencies demonstrated limited use of the JESIP multi-agency shared radio network.

Note: CCS = Casualty Clearing Station; CRU = Cycle Response Unit; DVI = Disaster Victim Identification; JESIP = Joint Emergency Services Interoperability Principles; LAS = London Ambulance Service NHS Trust; LFB = London Fire Brigade.

The observations brought to light a number of challenges with the processes followed. The initial tactical meeting (11:10) was very full, attended by multiple individuals from each agency, which meant that not all agencies could fit within the designated room. Moreover, there were inputs from different individuals from the same agency, prolonging the meeting. The police tactical commander addressed this by requesting a maximum of two people from each agency to attend the next meeting. Subsequent meetings consisted of the appropriate members from each agency, and LAS provided a progress update at the next meeting (12:00). It was noticeable that the inputs in the operational meeting at 17:31 consisted of repeated messages from earlier meetings, such as details of already known risks and on-site resources.

### The multi-agency response

A major incident requires a co-ordinated response from multiple agencies. This is because a major incident encompasses a number of characteristics that require different emergency services to work together, such as identifying the risks and appropriately resourcing the response. Successful co-ordination is primarily achieved through information sharing, which can be undertaken through a multi-agency shared radio network, up-to-date METHANE and IRIMAC reports and timely command meetings. An example of effective multi-agency working was outlined in Figure 3 (11:14), whereby the extraction of casualties was achieved by means of a ‘shuttle’. With the scene (platform) declared a HART/USAR (Urban Search and Rescue) response, the effectiveness of the casualty extraction relied on the early co-operation of LFB, USAR and HART teams.

**Figure 5:**
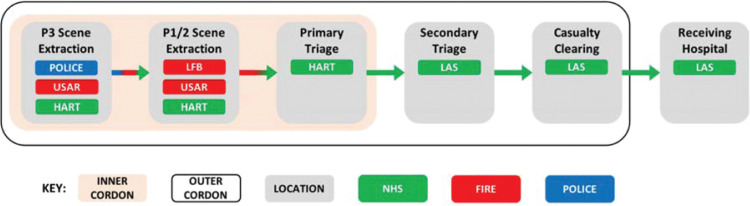
Figure 5. Multi-agency working at a critical incident: extraction to casualty clearing.

As demonstrated in Figure 5, the co-ordinated LFB, USAR and HART response to casualty extraction facilitated the transfer casualties from the HART-only area of the platform to the non-HART area. There, spare HART members undertook primary triage. Once triaged, casualties were extracted from the inner cordon by the police and continuously assessed by LAS responders (secondary triage) until reaching the CCS. The casualty’s departure from the CCS depended upon the next available ambulance, which then transferred casualties out of the outer cordon and to the receiving hospital.

## Discussion

The present service evaluation, focusing on LAS, recorded that LAS first responders to the EUR simulated incident rightly and rapidly declared the event a major incident, requested resources and assigned operational roles within the golden hour. Triage crews were tasked quickly, information was disseminated across the different agencies at a series of operational and tactical comment meetings and LAS together with LFB demonstrated effective multi-agency working by successfully co-ordinating their response with regards to casualty extraction.

A number of challenges were also noted, however, as a result of the observations. Firstly, within the ‘golden hour’, the number of casualties per triage priority level was not collected as a result of the initial briefing of triage crews not instructing them to collect such information. Casualty triage numbers would be useful information for the operational medic, whose job is to regularly update command with METHANE reports, which in turn impacts upon resourcing. Secondly, the initial tactical meeting was attended by multiple individuals from each agency, resulting in not all agencies fitting into the room. As a result, the inputs were not as focused as they could have been, causing a longer than necessary meeting. Finally, a later operational meeting consisted of repeated messages from earlier meetings, as all agencies were not making full use of the JESIP multi-agency shared radio network. Notably, with regards to risk, it was unlikely that an individual agency would be able to identify all risks on scene without information shared by other agencies.

Barriers to front line communication may adversely impact upon the reliability of the information collected, the sharing of such information and the quality of subsequent decisions (Nunan et al., 2016). In order to address these limitations, a number of areas for improvement can be identified. The efficiency of LAS triaging may be further developed by a greater use of communication within and across triage crews (observed to be lacking at 10:36), and more detailed triage briefings prior to entering the scene. In terms of improving the command meetings, a multi-agency METHANE report would have benefited the emergency response, as this would have highlighted gaps in each agency’s knowledge/progress of the incident and reduced the amount of repetition of information at meetings held later into day one. Thus, an encouraged use of the JESIP multi-agency talk-group would prevent the lack of communication which occurred due to agencies remaining in silos throughout the day, and minimise repeated messages at subsequent command meetings.

## Conclusion

The importance of successful multi-agency working cannot be overstated. Underpinning successful multi-agency working is the use of effective communication (Waring et al., 2017), which is implemented through information sharing (Allen et al., 2014), joint risk assessments and timely command meetings. Moreover, it is crucial that vital information such as the information elicited through METHANE and IRIMAC reports reaches the necessary LAS officer for it to be actioned. Recommendations arising from this service evaluation are that the JESIP multi-agency talk groups should be utilised more frequently and used to complete a joint METHANE report, as individual agencies working more closely together are likely to gather more comprehensive incident-relevant information.

In addition, training in areas such as communication skills, decision-making (see Power et al., 2018) and detailed briefings, supported using evidence-based tools, will enhance the front line response. The current project has shown that BWCs can be used as an effective service evaluation tool to examine real-world practices. BWCs are able to timestamp and capture both live time communication and behaviours during training exercises, which could be revisited and shown as training material or subjected to research analysis. Therefore, BWCs may also be useful in observing other areas of LAS practices (e.g. clinical skills, table-top exercises and real-world responses). The present evaluation has demonstrated that BWCs can promote best practice, identify training needs and highlight areas for future evaluation. Going forward, in order to successfully implement BWCs into training and research, academia and practitioners should continue to work together to generate impactful research and evaluations.

## Acknowledgements

The authors would like to thank the organisers of Exercise Unified Response for allowing the first author to attend and observe the simulation, as well the London Ambulance Service NHS Trust for providing privileged access to the data.

## Conflict of interest

None declared.

## Ethics

The University of Portsmouth’s Faculty of Humanities and Social Sciences granted favourable ethical approval for this service evaluation.

## Funding

This service evaluation was funded by the Faculty of Humanities and Social Sciences, University of Portsmouth.
